# Antioxidant Minerals Modified the Association between Iron and Type 2 Diabetes in a Chinese Population

**DOI:** 10.3390/nu16030335

**Published:** 2024-01-23

**Authors:** Teng Xu, Sitong Wan, Jiaxin Shi, Tiancheng Xu, Langrun Wang, Yiran Guan, Junjie Luo, Yongting Luo, Mingyue Sun, Peng An, Jingjing He

**Affiliations:** 1 College of Food Science and Nutritional Engineering, China Agricultural University, Beijing 100083, China; tengxu93@cau.edu.cn; 2 Key Laboratory of Precision Nutrition and Food Quality, Department of Nutrition and Health, Beijing Advanced Innovation Center for Food Nutrition and Human Health, China Agricultural University, Beijing 100190, China; adeline7wan@cau.edu.cn (S.W.); sy20233313705@cau.edu.cn (J.S.); sy20223313569@cau.edu.cn (L.W.); sy20233313730@cau.edu.cn (Y.G.); luojj@cau.edu.cn (J.L.); luo.yongting@cau.edu.cn (Y.L.); 3 School of Food and Health, Beijing Technology & Business University, Beijing 100048, China; 2102010328@st.btbu.edu.cn; 4 Xiyuan Hospital, China Academy of Chinese Medical Sciences, Beijing 100091, China

**Keywords:** type 2 diabetes, micronutrients, antioxidant mineral, nutritional intervention

## Abstract

Inconsistent findings exist regarding the relationship between heme iron intake and type 2 diabetes (T2D) among Western and Eastern populations. Easterners tend to consume a plant-based diet which is abundant in antioxidant minerals. To examine the hypothesis that antioxidant mineral may modify the relationship between iron and T2D, we performed a case–control study by measuring the serum mineral levels in 2198 Chinese subjects. A total of 2113 T2D patients and 2458 controls were invited; 502 T2D patients and 1696 controls were finally analyzed. In the total population, high serum iron showed a positive association with T2D odds (odds ratio [OR] = 1.27 [1.04, 1.55]); high magnesium (OR = 0.18 [0.14, 0.22]), copper (OR = 0.27 [0.21, 0.33]), zinc (OR = 0.37 [0.30, 0.46]), chromium (OR = 0.61 [0.50, 0.74]), or selenium concentrations (OR = 0.39 [0.31, 0.48]) were inversely associated with T2D odds. In contrast, in individuals with higher magnesium (>2673.2 µg/dL), zinc (>136.7 µg/dL), copper (>132.1 µg/dL), chromium (>14.0 µg/dL), or selenium concentrations (>16.8 µg/dL), serum iron displayed no association with T2D (*p* > 0.05). Serum copper and magnesium were significant modifiers of the association between iron and T2D in individuals with different physiological status (*p* < 0.05). Our findings support the idea that consuming a diet rich in antioxidant minerals is an effective approach for preventing T2D.

## 1. Introduction

Iron is an essential micronutrient for maintaining a variety of biological processes, including the regulation of the normal functions of pancreatic β cells [1]. Iron deficiency anemia is correlated with elevated blood glucose [2]. Nevertheless, iron also plays a key role in mediating the generation of reactive oxygen species via the Fenton reaction, thus causing oxidative stress, lipid peroxidation, and DNA damage in pancreatic β cells and the cells involved in glucose metabolism [1]. Excess iron intake or circulating iron are associated with an increased risk of type 2 diabetes (T2D) via modulating metabolic signaling pathways in the liver, pancreas, adipose tissue, and muscles [1,2].

Despite the fact that T2D is one of the common complications seen in hereditary hemochromatosis [3], an iron overload disease characterized by severe iron deposition in major organs, there is also direct evidence suggesting that iron is a risk determinant for T2D from studies of the iron storage marker ferritin in prospective cohorts. In healthy women from the Nurses’ Health Study, elevated ferritin concentration was associated with increased T2D incidence during 10 years of follow-up (relative risk [RR] = 2.68, 95% confidence intervals [CIs]: 1.75–4.11; highest vs. lowest quintile) [4]. A similar finding was reported in a 6-year follow-up survey of a Chinese prospective study (RR = 1.90, 95% CI: 1.37, 2.65; highest vs. lowest quintile) [5]. In a meta-analysis consisting of 15 prospective studies, elevated circulating ferritin increased in T2D incidence in a dose-dependent manner, and each 100 μg/L increment in circulating ferritin level was associated with a 22% increase in T2D risk [6].

In line with these findings, the intake of heme iron from red meat, but not non-heme iron, was identified to be positively associated with T2D incidence in participants from the Women’s Health Study, the Nurses’ Health Study, and the Health Professionals’ Follow-up Study [7,8,9]. The same conclusion was reached in a large prospective study involving Singapore Chinese populations [10]. However, contradictory findings were also reported in some prospective studies performed using Eastern populations, who tend to consume a plant-based diet characterized by a higher intake of vegetables and fruits and a lower intake of animal-based products. For example, no association was found between heme iron intake and T2D risk in a prospective analysis of participants from the China Health and Nutrition Survey (1991–2015) [11] and a prospective cohort in Japan [12]. Therefore, unrevealed modifying factors exist among the findings in prior cohort studies, and these factors may come from the differences in dietary pattern and ethnocultural background between Western and Eastern countries. The identification of the potential sources of heterogeneities will provide valuable references for dietary guidance to prevent T2D.

Plant-based diets are rich in antioxidant minerals such as magnesium and copper and contain relatively low levels of heme iron but higher levels of non-heme iron [13,14]. Antioxidant minerals, including but not limited to magnesium, selenium, and copper, are indispensable for maintaining the activity of several essential antioxidant enzymes, including glutathione peroxidases (GPXs) and superoxide dismutases (SODs) [15,16,17]. We proposed that the variation in serum antioxidant mineral levels may influence the relationship between iron and T2D. To validate our hypothesis that antioxidant minerals may serve as modifiers of the association between iron and T2D, we performed a case–control study by measuring the serum levels of multiple minerals (including magnesium, copper, zinc, selenium, manganese, and chromium) in 2198 Chinese residents in Shanghai city.

## 2. Materials and Methods

### 2.1. Study Population

The controls and T2D patients included in the present study were Shanghai residents that have been previously reported on [18,19,20,21]. From December 2006 to August 2007, based on a health registration system comprising local residents—developed by the Centers for Disease Control and Prevention (CDC) of Pudong and Baoshan Districts in Shanghai province (China)—a total of 2113 T2D patients and 2458 controls were recruited. Eligible persons were those who had lived in the local area for at least 5 years, those who were aged between 40 and 79 years, and those who were free of the following conditions: severe psychological disorders, dementia, physical disabilities, cancer, history of stroke, Alzheimer’s disease or coronary heart disease, tuberculosis, AIDS, or other communicable diseases. Anthropometric measurements were carried out by trained medical professionals or public health workers using a standardized protocol. Each participant provided informed consent. The study protocols were reviewed and approved by the ethics committee of the Shanghai Institute for Biological Sciences, Chinese Academy of Sciences. The present study adhered to the principles of the Declaration of Helsinki. Finally, a total of 502 T2D patients and 1696 control participants with physical and mineral measurements were selected as cases and controls, respectively, for this study.

### 2.2. Identification of T2D Cases and Controls

T2D patients were defined as those with fasting blood glucose (FBG) concentrations ≥ 7.0 mmol/L, in accordance with the World Health Organization criteria [22]. In this study, the T2D patients were initially identified from the health registration system in Pudong and Baoshan CDC; then, FBG was measured to further validate the presence of T2D before they were enrolled in the study. Th controls were residents recruited from the same communities as the T2D patients. The FBG concentrations of the control participants were <7.0 mmol/L.

### 2.3. Anthropometric and Biochemical Measurements

The anthropometric and fasting blood sample measurements were carried out as described previously [20,21]. In brief, body weight (in kilograms, kg), height (in centimeters, cm), waist circumference (cm), hip circumference (cm), and seated blood pressure (mmHg) were measured using a standardized protocol. Demographic information, including age and gender, was obtained through standardized questionnaires. FBG and other biochemical indicators (e.g., total cholesterol [TC], triglycerides [TG], low-density lipoprotein cholesterol [LDL-C], high-density lipoprotein cholesterol [HDL-C]) were measured using a Roche modular P800 autoanalyzer (Roche, Mannheim, Germany).

### 2.4. Serum Mineral Measurements

The preprocessing and measurements of the blood samples were carried out as described in a previous study [23]. An appropriate amount of serum samples was placed into a 15 mL centrifuge tube coated with PFA. After adding a certain amount of HNO_3_, the centrifuge tube was placed in a 150 °C water bath for 3 h. Then, the solution in the centrifuge tube was diluted with ultrapure water after clarification. Subsequently, the Agilent 7500cx Inductively Coupled Plasma Mass Spectrometry (ICP-MS) system (Agilent Technologies, Tokyo, Japan) was used in full quantitative mode to measure the mineral concentrations in the serum samples. To ensure the accuracy of the detection, quality control samples were tested after every 10 serum samples, and 10 parallel tests were conducted on each sample.

### 2.5. Statistical Analysis

First, the Kolmogorov–Smirnov test and a histogram were used to test the normal distribution of all continuous variables. According to the distributions, continuous variables were presented as mean ± standard deviations (for variables with normal distribution) or median (interquartile ranges) (for variables with skew distribution). Continuous variables were compared using the Mann–Whitney U test. Categorical variables were reported as counts (percentage) and were compared using the χ^2^ test. A binary logistic regression model was used to assess the potential modification of antioxidant minerals on the association between iron and T2D. All logistic regression analyses were adjusted for variables that could confound the modification of minerals on the relationship between iron and T2D. Thus, besides the crude Model 1, Model 2 was adjusted for age, gender, body mass index, TC, and TG. Each antioxidant mineral was classified as a binary variable by median as well as iron. We, separately determined the association between iron and T2D odds under a low or high level of copper, zinc, magnesium, selenium, manganese, and chromium. Subgroup analyses were conducted to estimate the potential impact on the results by age (≤60 or >60 years), gender (men or women), and body mass index (BMI, ≤24 or >24 kg/m^2^). Model 2 was selected as the main option for displaying the results of our subgroup analysis. All *p* values were two-sided, and *p* < 0.05 was considered statistically significant. All statistical analyses were performed using SPSS for Windows version 26.0 software (IBM Corporation, Chicago, IL, USA).

## 3. Results

### 3.1. Characteristics of the Controls and T2D Patients According to Serum Iron Levels

The baseline characteristics of the 2198 study participants are presented in Table 1. The mean (±SD) age of the total population was 57.99 ± 9.21 years. Compared with the controls, the T2D patients displayed higher BMI values, waist/hip circumference ratio values, SBP, TG, and FBG but lower HDL-C. Additionally, the T2D patients displayed higher serum iron concentrations but lower serum magnesium, copper, zinc, selenium, and chromium concentrations when compared with the controls.

### 3.2. Associations between Serum Mineral Concentrations and T2D

The independent associations between serum mineral concentrations (including iron and antioxidant minerals) and T2D are summarized in Table 2. High serum iron was positively associated with T2D in the crude model (Model 1, OR = 1.27 [1.04, 1.55]) and the model adjusted for age, gender, body mass index, TC, and TG (Model 2, OR = 1.24 [1.00, 1.52]). In both the crude model and the adjusted model, inverse associations with T2D were seen in high serum magnesium (OR = 0.18 [0.14, 0.23], Model 2), zinc (OR = 0.41 [0.33, 0.52], Model 2), chromium (OR = 0.66 [0.54, 0.82], Model 2), copper (OR = 0.29 [0.23, 0.37], Model 2), and selenium concentrations (OR = 0.44 [0.36, 0.55], Model 2).

### 3.3. Association between Serum Iron and T2D (Stratified by Antioxidant Mineral Concentrations)

To evaluate the potential modifying effect serum antioxidant minerals have on the relationship between iron and T2D, stratified analysis was performed using the median of each antioxidant mineral (Table 3). In individuals with low magnesium (≤2673.2 µg/dL), zinc (≤136.7 µg/dL), copper (≤132.1 µg/dL), chromium (≤14.0 µg/dL), or selenium levels (≤16.8 µg/dL), serum iron was positively associated with T2D odds in both the crude (Model 1) and the adjusted regression model (Model 2). In contrast, in individuals with high magnesium (>2673.2 µg/dL), zinc (>136.7 µg/dL), copper (>132.1 µg/dL), chromium (>14.0 µg/dL), or selenium levels (>16.8 µg/dL), serum iron displayed no association with T2D in both the crude and the adjusted model. Serum manganese level variation does not seem to be a significant modifier of the association between iron and T2D (*p* > 0.05).

As shown in Table 3, interactions may exist between magnesium and iron (*p*_interaction_ = 0.002) and between copper and iron (*p*_interaction_ = 0.077). Therefore, further analysis is needed to determine the potential modifying effect of these minerals on the association between iron and T2D.

### 3.4. Association between Iron and T2D (Stratified by Magnesium Levels According to Participants’ Physiological Status)

To investigate the interaction between iron and magnesium, we analyzed the association between iron and T2D via a further stratification according to participants’ physiological status (Table 4). The association between iron and T2D remains robust in populations with lower serum magnesium levels (≤2673.2 µg/dL), including in men (OR = 1.92 [1.20, 3.07]) and individuals with an age > 60 years old (OR = 1.48 [1.02, 2.16]) or BMI > 24 kg/m^2^ (OR = 1.49 [1.05, 2.10]). Notably, serum magnesium levels appear to modify the association between iron and T2D in individuals with BMI > 24 kg/m^2^ (*p*_interaction_ = 0.017).

### 3.5. Association between Iron and T2D (Stratified by Copper Levels According to Participants’ Physiological Status)

Next, we explored the potential interaction between iron and copper by employing further stratification according to participants’ physiological status to test the impact of copper on the association between iron and T2D odds (Table 5). The association between iron and T2D remains robust in populations with lower serum copper levels (≤132.1 µg/dL), including in men (OR = 1.90 [1.22, 2.97]), women (OR = 1.42 [1.01, 1.99]), individuals ≤ 60 years old (OR = 1.48 [1.01, 2.12]), individuals > 60 years old (OR = 1.73 [1.18, 2.53]), individuals with BMI ≤ 24 kg/m^2^ (OR = 1.71 [1.13, 2.59]), and individuals with BMI > 24 kg/m^2^ (OR = 1.54 [1.08, 2.19]). Moreover, serum copper levels appear to modify the association between iron and T2D in men (*p*_interaction_ = 0.029), women (*p*_interaction_ = 0.042), individuals > 60 years old (*p*_interaction_ = 0.016), and individuals with BMI > 24 kg/m^2^ (*p*_interaction_ = 0.007).

## 4. Discussion

Inconsistent findings exist regarding the relationship between heme iron intake and T2D risk among studies in Western and Eastern populations [11]. Easterners, especially Chinese people, tend to consume a plant-based diet which is abundant in antioxidant micronutrients. We proposed that antioxidant minerals may be potential modifiers of the association between iron and T2D in Chinese populations. By measuring serum total iron and multiple antioxidant mineral levels, we investigated the association between serum iron and T2D in individuals with different levels of antioxidant minerals. Serum magnesium, copper, zinc, chromium, and selenium concentrations were shown to be inversely associated with the odds of developing T2D among Chinese urban residents (Table 2). Interestingly, the association between iron and T2D were only seen in individuals with lower levels of these antioxidant minerals (Table 3). Our results also suggest that magnesium and copper may have interactions with iron via significantly modifying the iron-T2D association in individuals with different physiological status (Table 4 and Table 5).

In this study, the T2D patients had higher serum iron levels than the controls (median 581.99 µg/dL vs. 536.33 µg/dL, *p* < 0.001; Table 1), and higher serum iron (>544.6 µg/dL) is associated with increased T2D odds (Table 2). The exact mechanism by which excess iron intake and iron stores promote the pathogenesis of T2D remains to be elucidated. Evidence from several studies may provide insight into the role of iron in glucose and energy metabolism. Excessive iron may cause excessive oxidative stress in pancreatic β cells. In a murine model of hemochromatosis, iron deposition decreased insulin secretory capacity secondary to oxidative stress, and increased apoptosis in pancreatic β-cells [24]. In addition, mechanistic studies have also indicated that multiple metabolic signaling pathways could be affected by iron levels, including those responsible for glucose homeostasis, appetite regulation, and hormone secretion [1]. For example, dietary iron upregulated fasting glucose concentrations via increasing liver glucose output [25]. Dietary iron directly increased food intake by negatively regulating leptin transcription in adipose tissue [26]. In the adipocytes, increased iron also induced a less expressed adiponectin, an insulin-sensitizing adipokine [27].

Another important influence of iron on the development of T2D involves the perturbation of cellular stress response. Iron was found to impede the intracellular trafficking of several important antioxidant minerals. In iron overload mice, excess iron accumulation inhibited the mitochondrial uptake of copper, manganese, and zinc, resulting in a decrease in the activity of copper-dependent cytochrome c oxidase and manganese-dependent superoxide dismutase (MnSOD) [28]. Besides these molecules, several additional antioxidant molecules presented in tissues also require these minerals to maintain their capacity for eliminating excessive reactive oxygen species under stress [29,30]. For example, to alleviate oxidative damage outside of the mitochondria, copper and zinc ions are needed for cytoplasmic SOD (Cu/ZnSOD) to catalyze the disproportionation of superoxide [31]. Selenium is required for glutathione peroxidases (GPXs), which eliminate peroxides in the cytosol and mitochondria by utilizing glutathione [29]. The antioxidant activities of SODs and GPXs were found to be dependent on intracellular magnesium levels [15].

In large surveys conducted in China, such as the China National Nutrition and Health Survey, an inadequate intake of magnesium, zinc, selenium, and manganese were identified in Chinese populations [32,33]. It is estimated that 45.8% of elderly Chinese people have an insufficient dietary intake of zinc, and >50% of Chinese children and >80% of elderly Chinese people are estimated to have an insufficient dietary intake of selenium [32]. In our study, the T2D patients displayed lower serum magnesium, copper, zinc, selenium, and chromium concentrations than the controls (Table 1). Higher antioxidant mineral concentrations, including for minerals such as magnesium (>2673.2 µg/dL), copper (>132.1 µg/dL), zinc (>136.7 µg/dL), chromium (>14.0 µg/dL), and selenium (>16.8 µg/dL), were negatively associated with T2D. These findings support previous reports stating that the dietary intake of higher antioxidant minerals protects against T2D risk. Increased dietary magnesium intake was shown to be associated with a reduced risk of T2D in a meta-analysis of 40 prospective cohort studies [34]. Increased manganese intake was shown to be inversely associated with T2D risk in prospective studies performed in the US [35] and China [36]. Zinc supplementation decreased fasting blood glucose, hemoglobin A1c, and fasting blood insulin in a meta-analysis of 20 interventional studies performed in healthy persons and individuals with metabolic diseases [37]. Chromium supplementation was shown to improve multiple blood glucose parameters, especially in populations from non-Western countries [38].

To the best of our knowledge, we are the first to report that mineral status is a modifier of the association between iron and T2D. The mechanism by which antioxidant minerals modify the T2D risk brought by iron may come from two aspects. First of all, increased antioxidant minerals improve overall cardiometabolic health due to their capacity to eliminate reactive oxygen species and inflammation. Magnesium supplementation significantly reduced human inflammatory markers such as C-reactive protein in a meta-analysis of 17 interventional trials [39]. In a meta-analysis of 25 interventional trials, zinc supplementation displayed anti-inflammatory and antioxidative effects in adults [40]. Plasma manganese levels and their interaction with oxidative stress markers have been proposed as mediators in the association between dietary manganese intake and T2D risk in prospective studies [35,36]. However, in our analysis, we did not observe a significant association between manganese and T2D odds (OR = 1.18 [0.96, 1.45], Model 2). This is probably due the differences in serum manganese levels among Chinese people and people from different geographical locations. The median serum manganese level reported in this study (2.34 µg/L, Table 1) is higher than the median serum manganese levels in a nation-wide survey performed in China (males: 1.60 µg/L; females:1.51 µg/L) [41], which suggests that the participants of the current study had sufficient serum manganese levels. Regarding the role of copper in T2D, a unique finding was noted in our study. In a prior case–control study of adults from nine provinces of China, serum copper levels showed no influence on T2D risk [42]; in contrast, our analysis identified an inverse association between serum copper and T2D (OR = 0.29 [0.23, 0.37]) (Table 2). Compared with this previous study, the participants in this study were all stable residents from communities in Shanghai’s urban areas, which may have minimized the heterogeneity among the study population. In addition, our analysis also identified that higher serum copper (>132.1 µg/L) modified the association between serum iron and T2D (Table 5).

The other possible mechanism through which antioxidant minerals modified the association between iron and T2D could be attributable to the antagonistic effect of divalent metal ions, because they share the same intestinal absorption transporter, divalent metal transporter 1 (DMT1) [43]. In one study, the simultaneous supplementation of iron and zinc displayed a lower iron supplemental effect than the supplementation of iron alone in pregnant women, and women who received iron supplementation were shown to have lowered serum zinc levels [44]. Similarly, infants receiving iron supplementation were characterized by decreased manganese concentrations [45]. Absorptive interactions between iron and chromium, as well as iron and selenium, have also been reported in studies using cell or animal models [46].

This study has several limitations, including some related to the study design and the analyses carried out in the current study. First, this study had a case–control design, which limited the exploration of the causal relationship between serum mineral levels and T2D. Second, some variables were not recorded in the present study. For example, information on smoking and drinking habits were not available for all participants. Therefore, this impeded the inclusion of these variables as confounders in our analyses. In addition, the therapeutic information of each T2D patient was not recorded, and thus the potential impact of blood glucose-, blood pressure- and blood lipid-lowering therapeutics on serum mineral levels could not be evaluated. Third, the participants recruited for this study were residents from a big city. The conclusions obtained from this study cannot be directly generalized to individuals living in the rural areas of China due to differences in dietary pattern and socioeconomic status. Dietary mineral intake is significantly different between big cities and rural areas, as well as between North China and South China, according to the China National Nutrition and Health Survey [32].

## 5. Conclusions

The concentration of serum antioxidant minerals modified the association between iron and T2D in Chinese urban residents. Higher levels of serum magnesium or copper were associated with lower odds of developing T2D brought by iron in middle-aged and elderly Chinese persons. The results from this study support the idea that consuming a diet that is rich in antioxidant minerals can serve as an effective approach for preventing T2D. Additionally, our findings may also provide a reasonable explanation for the heterogeneity observed in the relationship between heme iron intake and T2D risk among populations with different dietary patterns.

## Figures and Tables

**Table 1:** Characteristics of the study population.

	Control (n = 1696)	Type 2 Diabetes (n = 502)	Total (n = 2198)	*p*
Age (year)	56.97 ± 9.09	61.47 ± 8.77	57.99 ± 9.21	<0.001
Gender (n, %)				
Male	572 (33.7)	200 (39.8)	772 (35.3)	<0.001
Female	1124 (66.3)	302 (60.2)	1426 (64.7)	
BMI, kg/m^2^	24.35 ± 3.08	24.87 ± 3.28	24.47 ± 3.14	0.002
Waist–hip ratio	0.85 ± 0.06	0.89 ± 0.07	0.86 ± 0.07	<0.001
SBP, mmHg	130.71 ± 17.79	140.97 ± 22.43	133.08 ± 19.44	<0.001
DBP, mmHg	80.74 ± 9.74	80.93 ± 10.30	80.78 ± 9.87	0.661
HDL-C, mmol/L	1.34 ± 0.33	1.17 ± 0.32	1.30 ± 0.34	<0.001
LDL-C, mmol/L	2.65 ± 0.63	2.67 ± 0.72	2.65 ± 0.65	0.969
TC, mmol/L	4.81 ± 0.94	4.64 ± 1.05	4.77 ± 0.97	<0.001
TG, mmol/L	1.68 ± 1.26	2.05 ± 1.69	1.76 ± 1.38	<0.001
FBG, mmol/L	4.66 ± 0.63	8.41 ± 2.85	5.52 ± 2.15	<0.001
Magnesium, µg/L	2803.77 (2088.50, 3114.37)	1564.20 (983.14, 2459.84)	2673.25 (1315.45, 3048.25)	<0.001
Copper, µg/dL	137.75 ± 40.23	113.95 ± 35.92	132.09 ± 40.63	<0.001
Zinc, µg/dL	146.55 ± 83.84	124.52 ± 47.02	141.52 ± 77.54	<0.001
Chromium, µg/dL	15.95 (6.77, 37.68)	8.39 (2.56, 28.65)	13.97 (5.80, 35.24)	<0.001
Selenium, µg/dL	17.90 (12.95, 24.59)	13.83 (11.43, 19.54)	16.81 (12.37, 23.08)	<0.001
Manganese, µg/dL	2.32 (1.45, 3.96)	2.42 (1.40, 4.16)	2.34 (1.45, 4.00)	0.545
Iron, µg/dL	536.33 (398.60, 743.12)	581.99 (428.57, 942.91)	544.56 (403.56, 768.99)	<0.001

Values are presented as mean ± standard deviations, median (interquartile ranges), or count (%). The Mann–Whitney U test was used to compare the continuous variables. The χ^2^ test was used for comparing the categorical variables. BMI, body mass index; SBP, systolic blood pressure; DBP, diastolic blood pressure; HDL-C, high-density lipoprotein cholesterol; LDL-C, low-density lipoprotein cholesterol; TC, total cholesterol; TG, triglycerides; FBG, fasting blood glucose. *p* values were obtained from the comparisons between the control participants and type 2 diabetes patients.

**Table 2:** Independent associations between serum minerals and type 2 diabetes.

Serum Minerals	N (%)	Case (%)	Model 1	Model 2
Iron, µg/dL				
≤544.6	1099 (50.0)	228 (20.8)	1 (reference)	1 (reference)
>544.6	1099 (50.0)	274 (24.9)	1.27 (1.04, 1.55)	1.24 (1.00, 1.52)
*p*			0.020	0.047
Magnesium, µg/dL				
≤2673.2	1099 (50.0)	401 (36.5)	1 (reference)	1 (reference)
>2673.2	1099 (50.0)	101 (9.2)	0.18 (0.14,0.22)	0.18 (0.14,0.23)
*p*			<0.001	<0.001
Copper, µg/dL				
≤132.1	1099 (50.0)	371 (33.7)	1 (reference)	1 (reference)
>132.1	1099 (50.0)	131 (11.9)	0.27 (0.21, 0.33)	0.29 (0.23, 0.37)
*p*			<0.001	<0.001
Zinc, µg/dL				
≤136.7	1099 (50.0)	343 (31.2)	1 (reference)	1 (reference)
>136.7	1099 (50.0)	159 (14.5)	0.37 (0.30, 0.46)	0.41 (0.33, 0.52)
*p*			<0.001	<0.001
Chromium, µg/dL				
≤14.0	1099 (50.0)	299 (27.2)	1 (reference)	1 (reference)
>14.0	1099 (50.0)	203 (18.5)	0.61 (0.50, 0.74)	0.66 (0.54, 0.82)
*p*			<0.001	<0.001
Selenium, µg/dL				
≤16.8	1099 (50.0)	340 (30.9)	1 (reference)	1 (reference)
>16.8	1099 (50.0)	162 (14.6)	0.39 (0.31, 0.48)	0.44 (0.36, 0.55)
*p*			<0.001	<0.001
Manganese, µg/dL				
≤2.3	1099 (50.0)	241 (21.9)	1 (reference)	1 (reference)
>2.3	1099 (50.0)	261 (23.7)	1.11 (0.91, 1.35)	1.18 (0.96, 1.45)
*p*			0.310	0.125

Odds ratios (95% confidence intervals) and *p* values were obtained from the logistic regression models. Model 1—crude model; Model 2—model further adjusted for age, gender, body mass index, total cholesterol, and triglycerides.

**Table 3:** Association between serum iron and type 2 diabetes (stratified by serum antioxidant mineral concentrations).

			Model 1		Model 2	
Serum Minerals	N (%)	Case (%)	OR (95% CI)	*p*	OR (95% CI)	*p*
Magnesium, µg/dL						
≤2673.2	1099 (50.0)	401 (36.5)	1.44 (1.12, 1.84)	0.004	1.42 (1.10, 1.85)	0.008
>2673.2	1099 (50.0)	101 (9.2)	0.99 (0.65, 1.48)	0.940	0.91 (0.60, 1.39)	0.665
*p* for interaction				0.004		0.077
Copper, µg/dL						
≤132.1	1099 (50.0)	371 (33.7)	1.61 (1.25, 2.07)	<0.001	1.62 (1.24, 2.11)	<0.001
>132.1	1099 (50.0)	131 (11.9)	0.80 (0.56, 1.16)	0.240	0.77 (0.53, 1.12)	0.164
*p* for interaction				<0.001		0.002
Zinc, µg/dL						
≤136.7	1099 (50.0)	343 (31.2)	1.57 (1.22, 2.03)	0.001	1.57 (1.20, 2.06)	0.001
>136.7	1099 (50.0)	159 (14.5)	1.23 (0.87, 1.72)	0.245	1.15 (0.81, 1.63)	0.448
*p* for interaction				0.255		0.150
Chromium, µg/dL						
≤14.0	1099 (50.0)	299 (27.2)	1.67 (1.27, 2.19)	<0.001	1.59 (1.19, 2.12)	0.002
>14.0	1099 (50.0)	203 (18.5)	1.36 (0.98, 1.90)	0.066	1.32 (0.94, 1.86)	0.111
*p* for interaction				0.358		0.428
Selenium, µg/dL						
≤16.8	1099 (50.0)	340 (30.9)	1.35 (1.04, 1.74)	0.023	1.33 (1.02, 1.75)	0.039
>16.8	1099 (50.0)	162 (14.6)	0.99 (0.71, 1.38)	0.936	0.96 (0.68, 1.36)	0.818
*p* for interaction				0.146		0.224
Manganese, µg/dL						
≤2.3	1099 (50.0)	241 (21.9)	1.24 (0.92,1.66)	0.154	1.28 (0.94, 1.74)	0.116
>2.3	1099 (50.0)	261 (23.7)	1.27 (0.95, 1.71)	0.111	1.11 (0.82, 1.52)	0.500
*p* for interaction				0.898		0.635

Odds ratios (95% confidence intervals) and *p* values were obtained from the logistic regression models. Serum iron was included in the analysis as a binary variable according to the median, with the lower group serving as the reference group. Model 1—crude model; Model 2—model further adjusted for age, gender, body mass index, total cholesterol, and triglycerides. *p*-values for interaction were assessed using a likelihood ratio test to compare the models with and without interaction terms. OR—odds ratio; CI—confidence interval.

**Table 4:** Association between serum iron and type 2 diabetes (stratified by magnesium levels according to physiological status).

		N	Case (%)	OR (95%CI)	*p*	*p*_interaction_
Age, years	Magnesium, µg/dL					
≤60	≤2673.2	645	169 (26.2)	1.34 (0.92, 1.94)	0.128	0.201
	>2673.2	712	64 (9.0)	0.91 (0.54, 1.53)	0.727	
>60	≤2673.2	454	232 (51.1)	1.48 (1.02, 2.16)	0.039	0.332
	>2673.2	387	37 (9.6)	0.80 (0.38, 1.70)	0.567	
Gender						
Men	≤2673.2	345	152 (44.1)	1.92 (1.20, 3.07)	0.006	0.073
	>2673.2	427	48 (11.2)	0.95 (0.51, 1.78)	0.870	
Women	≤2673.2	754	249 (33.0)	1.16 (0.84, 1.61)	0.358	0.383
	>2673.2	672	53 (7.9)	0.88 (0.49, 1.57)	0.661	
BMI, kg/m^2^						
≤24	≤2673.2	488	157 (32.2)	1.35 (0.90, 2.02)	0.145	0.989
	>2673.2	514	39 (7.6)	1.26 (0.65, 2.46)	0.491	
>24	≤2673.2	611	244 (40.0)	1.49 (1.05, 2.10)	0.024	0.017
	>2673.2	585	62 (10.6)	0.70 (0.41, 1.21)	0.198	

Odds ratios (95% confidence intervals) and *p* values were obtained from the logistic regression model adjusted for age, gender, body mass index, total cholesterol, and triglycerides. Serum iron was included in the analysis as a binary variable according to the median, with the lower group serving as the reference group. *p*-values for interaction were assessed using a likelihood ratio test to compare the models with and without interaction terms. BMI, body mass index; OR, odds ratio; CI, confidence interval.

**Table 5:** Association between serum iron and type 2 diabetes (stratified by copper levels according to physiological status).

		N	Case (%)	OR (95%CI)	*p*	*p*_interaction_
Age, years	Copper, µg/dL					
≤60	≤132.1	655	149 (22.7)	1.48 (1.01, 2.12)	0.046	0.054
	>132.1	702	84 (11.9)	0.82 (0.51, 1.30)	0.393	
>60	≤132.1	444	222 (50.0)	1.73 (1.18, 2.53)	0.005	0.016
	>132.1	397	47 (11.8)	0.63 (0.33, 1.23)	0.175	
Gender						
Men	≤132.1	408	153 (37.5)	1.90 (1.22, 2.97)	0.004	0.029
	>132.1	364	47 (12.9)	0.75 (0.39, 1.45)	0.398	
Women	≤132.1	691	218 (31.5)	1.42 (1.01, 1.99)	0.046	0.042
	>132.1	735	84 (11.4)	0.78 (0.49, 1.24)	0.288	
BMI, kg/m^2^						
≤24	≤132.1	483	152 (31.5)	1.71 (1.13, 2.59)	0.011	0.079
	>132.1	519	44 (8.5)	0.82 (0.43, 1.54)	0.528	
>24	≤132.1	616	219 (35.6)	1.54 (1.08, 2.19)	0.017	0.007
	>132.1	580	87 (15.0)	0.69 (0.43, 1.11)	0.125	

Odds ratios (95% confidence intervals) and *p* values were obtained from the logistic regression model adjusted for age, gender, body mass index, total cholesterol, and triglycerides. Serum iron was included in the analysis as a binary variable according to the median, with the lower group serving as the reference group. *p*-values for interaction were assessed using a likelihood ratio test to compare the models with and without interaction terms. BMI, body mass index; OR, odds ratio; CI, confidence interval.

## Data Availability

The raw data supporting the conclusions of this article will be made available by the authors without undue reservation.
